# Co-Supplementation of Policosanol and Atorvastatin Attenuates High-Cholesterol and High-Galactose Diet-Induced Ocular and Brain Damage and Improves Cognitive Health in Zebrafish

**DOI:** 10.3390/ph19101622

**Published:** 2026-10-13

**Authors:** Kyung-Hyun Cho, Ashutosh Bahuguna, Seung Hee Baek, Chae-Eun Yang

**Affiliations:** Raydel HDL Research Institute, Medical Innovation Complex, Daegu 41061, Republic of Korea

**Keywords:** brain, cognitive test, cornea, myelin, oxidative stress, retina, senescence, somites

## Abstract

**Objectives:** This study compares the effects of policosanol (PCO), atorvastatin (ATV), and their combination (ATV + PCO) on high-cholesterol and high-galactose diet (HCHG)-induced adverse events in the eyes and brain, as well as cognitive behavior, in zebrafish. **Methodology:** Adult zebrafish (*n* = 42/group) were fed for 22 weeks with HCHG, or HCHG co-supplemented with PCO, ATV, or ATV + PCO. Corneal clouding was assessed in all the groups, while retina and brain tissue were collected for histological and immunohistochemical analyses. Cognitive behavior across the groups was analyzed using the open field test, and memory impairment was assessed using electric pulse (passive avoidance) response tests. **Results:** After 22 weeks of feeding, the highest corneal clouding, with an opacity index of 1.1 ± 0.16, was observed in the HCHG group, which was substantially reduced by 2.2-fold and 1.9-fold by the supplementation of PCO and ATV + PCO, respectively. The ocular histological analysis revealed a significant 3.9-fold and 2.1-fold lower reactive oxygen species (ROS) generation in the retina of PCO- and ATV + PCO-supplemented zebrafish, compared with the HCHG-fed zebrafish. Consistently, brain histology suggests substantially lower ROS generation and apoptosis in the tectum opticum (TeO) and periventricular gray zone (PGZ) of the PCO and ATV + PCO groups than in the HCHG group. Likewise, PCO and ATV + PCO supplementation substantially prevented HCHG-induced senescence and demyelination in the brain. HCHG-induced cognitive impairment, marked by a reduced swimming speed, distance, and geotaxis swimming pattern (bottom dwelling), was substantially improved with co-supplementation of PCO and ATV + PCO. Furthermore, the PCO- and ATV + PCO-supplemented groups showed reduced confusion and higher escape capacity in response to electric pulses during the passive avoidance test. **Conclusions:** PCO and ATV + PCO supplementation provided a beneficial effect against HCHG-induced ocular and brain damage by mitigating oxidative stress, apoptosis, senescence, and demyelination, and simultaneously improving cognitive and memory decline in zebrafish.

## 1. Introduction

Excessive galactose consumption leads to galactitol formation and catalyzes the formation of non-enzymatic advanced glycation end products (AGEs), which adversely affect a variety of organs, including the brain [1,2,3]. Galactitol impairs cellular antioxidants, induces reactive oxygen species (ROS) production, damages a variety of macromolecules, and alters cellular membrane integrity [1,4]. Likewise, AGEs also have several pathological outcomes, primarily facilitated through interaction with the receptor for advanced glycation end products (RAGE), which activates the nuclear factor kappa B (NFκB) pathway and induces inflammation and oxidative stress [5]. In addition, AGE/RAGE association modulates the gene expression profile responsible for the advancement of type 2 diabetes [5]. High galactose intake induces cellular senescence, oxidative stress, and inflammation, leading to premature aging [4]. The notable effects of high exposure to galactose on cognitive impairment, neurodegeneration, and cellular damage to the eyes, resulting in severe complications like cataract and blindness, have been well established [6]. High cholesterol and high-fat intake induce metabolic stress and adversely affect the function of different organs [7,8,9]. High cholesterol consumption has been associated with impaired antioxidant status and increased oxidative stress. Although high cholesterol is primarily linked to atherosclerosis [10], accumulating evidence suggests that it may also contribute to the development and progression of several other diseases, including renal dysfunction, liver disease, osteoporosis, osteoarthritis, diabetes, and Alzheimer’s disease [11]. The co-exposure to high cholesterol and high galactose served as a dietary model to induce metabolic stress and organ damage [12].

Policosanol is a blend of long-chain aliphatic alcohols (LCAA) widely recognized for its cholesterol-lowering effect [13,14]. In addition, policosanol has been shown to improve high-density lipoprotein (HDL) function [15,16], enhance cholesterol efflux capacity [17], prevent oxidative injury to low-density lipoprotein (LDL), and exert anti-glycation effects [16]. Additionally, studies have deciphered the beneficial effects of policosanol on ischemic heart disease, hypertension, and neurological disorders [13,14]. Policosanol can be obtained from various natural sources, and its composition may differ considerably depending on the source and extraction process [18]. For instance, policosanol extracted from Cuban sugarcane wax is a typical mixture of eight characteristic LCAAs (C_24_-C_34_) [19], and its composition and functionality vary substantially from other policosanols [20]. Notably, in Korea, only policosanol sourced from Cuban sugarcane wax is approved by the Ministry of Food and Drug Safety (MFDS) as a functional food for health promotion activities [21]. 

Atorvastatin is a commonly used statin that treats dyslipidemia primarily by inhibiting 3-hydroxy-3-methylglutaryl-coenzyme A (HMG-CoA) reductase, a key rate-limiting enzyme in cholesterol biosynthesis [22]. Beyond its lipid-lowering effects, atorvastatin has been shown to improve endothelial function, reduce platelet aggregation, stabilize atherosclerotic plaques, and exert anti-inflammatory effects [23,24]. Besides the beneficial effects, a prolonged intake of statins, including atorvastatin, has undesirable effects on different organs [25,26,27,28] and can cause peripheral neuropathy [29]. The existing literature presents conflicting evidence regarding the effect of statins on cognitive decline in humans [30]. The available literature suggests atorvastatin has no modulatory effect on the eyes; however, a case study reports visual disturbance in response to atorvastatin [31].

Researchers have extensively investigated the individual effects of policosanol and atorvastatin on metabolic stress. However, the comparative evaluation of the effects of these two, particularly their combination on eyes and brain health and cognitive function, remains largely unexplored. In our previous study, we evaluated the effects of 0.1% policosanol (PCO) and atorvastatin (ATV), alone and in combination (ATV + PCO), after 12 weeks of feeding in hyperlipidemic, hyperglycemic zebrafish [32]. In another previous study, lower amounts (0.05%) of PCO, ATV, and ATV + PCO were investigated for their effects on dyslipidemia, blood glucose, biochemical parameters, tissue regeneration, and histological changes in the liver, kidney, intestine, and reproductive organs of hyperlipidemic, hyperglycemic zebrafish [33]. Nevertheless, the previous study [33] did not investigate eye and brain health, cognitive and memory functions, or the functionality of plasma against external stress. The present study is an extension of our previous work [33] by using the same cohorts of zebrafish (*Danio rerio*) exposed to a 22-week high-cholesterol and high-galactose (HCHG) diet to evaluate the effects of 0.05% PCO, ATV, and ATV + PCO supplementation on eye and brain health, as well as cognitive and memory function. In addition, the plasma collected from these zebrafish was evaluated for its functionality against carboxymethyllysine (CML)-induced external stress using zebrafish embryos.

Zebrafish were selected as a model organism because their genome closely resembles humans [34] and many pathological processes progress similarly to those in humans [35]. The small size, rapid development, and ease of husbandry of zebrafish make it easy to maintain large numbers in a relatively small space. Zebrafish eye development and essential eye components are highly similar to those of humans [36]. Similar to humans, zebrafish have comparable corneal and lens morphology and are therefore considered a good experimental model for examining disorders such as cataract and diabetic retinopathy [36]. The zebrafish brain also closely resembles human neuroanatomical pathways [37] and thus serves as a good model for various human brain disorders and behavioral changes [38].

## 2. Results

### 2.1. Swimming Behavior Analysis of Adult Zebrafish

The open-field tank swimming analysis revealed a substantial variation in swimming distance and swimming pattern between groups (Figure 1). The swimming trajectories of the ND (control) group showed a normal swimming pattern, with zebrafish moving freely across the tank in both horizontal and vertical directions (Figure 1A,B). In contrast, zebrafish in the HCHG group showed severely impaired swimming ability. Zebrafish in the HCHG group were mainly restricted to the lower half of the tank, and many fish showed geotaxis behavior (swimming along the bottom). In contrast, zebrafish in the HCHG co-supplemented with PCO group showed normal swimming patterns, with free access to the top and bottom of the tank. Zebrafish in the HCHG co-supplemented with ATV group showed impaired swimming, mainly restricted to the lower half of the tank. In contrast, the HCHG co-supplemented with ATV + PCO group showed free-swimming movement across the tank.

The quantitative outcome of the swimming analysis revealed a significantly highest swimming distance (19.7 ± 0.39 m) and percentage stayed time in the upper half (42.8 ± 0.89%) that was significantly 13.2-fold (*p* < 0.001) and 23.7-fold (*p* < 0.001) higher than the swimming distance (1.5 ± 0.16 m), and stayed in the upper half (1.8 ± 0.42%) of the tank than the HCHG group (Figure 1C,D). Compared to the HCHG group, the zebrafish in the PCO-, ATV-, and ATV + PCO-supplemented groups showed a significantly 13.1-fold (*p* < 0.001) 3.6-fold (*p* = 0.01), and 10.5-fold (*p* < 0.001) higher swimming distance and 19.3-fold (*p* < 0.001), 5.2-fold (*p* < 0.001), and 13.1-fold (*p* < 0.001) higher stay time in the upper half, respectively. Compared with the ATV-supplemented group, the ATV + PCO group showed a notable 2-fold (*p* = 0.000) and 3-fold (*p* = 0.000) higher swimming distance and stay time in the upper half.

### 2.2. Passive Avoidance Response in Zebrafish

The findings of the passive avoidance test revealed a significantly 10.6-fold (*p* < 0.001) reduced frequency of boundary crossing (between the dark and light chambers) and a 7.3-fold (*p* < 0.001) higher stay time on the light side of the tanks for the zebrafish in the ND control group compared to the HCHG group (Figure 2A–D). The HCHG co-supplemented with PCO and ATV + PCO showed a significant 8-fold (*p* < 0.001) and 5.3-fold (*p* < 0.001) lower frequency of boundary crossing (between the dark and light chambers) and 7.1-fold (*p* < 0.001) and 6.3-fold (*p* < 0.001) higher stay time on the light side of the tank, respectively, compared to the HCHG group. Interestingly, ATV supplementation did not significantly affect zebrafish frequency of boundary crossing or stay time on the light side of the tank compared with the control group.

The findings underscore the substantial effects of PCO and ATV + PCO on HCHG-induced alterations in zebrafish swimming behavior and threat avoidance tendencies.

### 2.3. Brain Histology

H&E staining of the brain, as shown in Figure 3A,B, revealed a minor morphological change in the HCHG group. Compared with the ND (control) group, the HCHG group showed a high prevalence of mononuclear cells with clear zones and vacuolation in the tectum opticum (TeO) and periventricular gray zone (PGZ). In contrast, the brain sections from the PCO-, ATV-, and ATV + PCO-supplemented groups showed less vacuolation and fewer mononuclear cells with clear zones in the TeO and PGZ regions.

DHE and AO staining correspond to ROS, and apoptosis showed the highest fluorescence intensity around the TeO and PGZ, mainly accumulating in the valvular cerebelli (Val) region of the brain in the HCHG-supplemented group (Figure 3C,D,H,I). Compared with the ND (control) group, the HCHG-supplemented group showed significantly higher ROS and apoptosis (8.1-fold and 6.6-fold, respectively). ATV supplementation did not significantly affect HCHG-induced ROS or apoptosis. In contrast, PCO and ATV + PCO supplementation significantly reduced ROS generation by 4.6-fold (*p* < 0.01) and 3.3-fold (*p* < 0.01), and apoptosis by 4.5-fold (*p* < 0.01) and 3.7-fold (*p* < 0.01), respectively.

IHC staining of the brain shows the highest (3.8 ± 0.4 × 10^3^ AU) myelin-specific fluorescent intensity in the ND (control) group, which was 8.2-fold (*p* < 0.001) higher than the myelin-related fluorescent intensity (0.46 ± 0.1 × 10^3^ AU) observed in the HCHG-supplemented group (Figure 3E,J). HCHG-induced demyelination (reflected by the low myelin-related fluorescent intensity) was enhanced to 2.2 ± 0.1 × 10^3^ AU and 1.5 ± 0.2 × 10^3^ AU with PCO and ATV + PCO supplementation, respectively, and was significantly 4.6-fold (*p* < 0.01) and 3-fold (*p* < 0.05) higher than the myelin-specific fluorescent intensity observed in the HCHG group.

The SA-β-gal staining suggests the highest prevalence of SA-β-gal-positive cells (5.6 ± 0.4%) in the brain of the HCHG group compared to the ND group (1.4 ± 0.3%) (Figure 3F,G,K). The HCHG-induced prevalence of senescent positive cells was substantially reduced by 2.8-fold (*p* < 0.001), 1.4-fold (*p* < 0.01), and 2.2-fold (*p* < 0.001) by the supplementation of PCO, ATV, and ATV + PCO, respectively. Notably, compared with ATV alone, the ATV + PCO-supplemented group showed a significant 1.6-fold (*p* = 0.004) lower number of senescent-positive cells, underscoring the greater efficacy of ATV + PCO in preventing senescence than ATV.

### 2.4. Changes in Cornea and Iris

In most zebrafish eyes, severe corneal clouding (opacity index 1.1 ± 0.16) appeared after 22 weeks of supplementation with HCHG, which was notably 4.6-fold (*p* < 0.001) higher than the opacity index (0.24 ± 0.09) detected in the eyes of zebrafish consuming an ND diet (Figure 4A,B). Likewise, a high corneal opacity index (1.03 ± 0.12) was observed in the eyes of the HCHG co-supplemented with ATV group. In contrast, a significantly lower corneal clouding, with opacity indices of 0.50 ± 0.10 and 0.59 ± 0.11, was observed in the HCHG co-supplemented with PCO and ATV + PCO groups, respectively, which is notably 2.2-fold (*p* < 0.01) and 1.9-fold (*p* < 0.05) lower, respectively, than the corneal opacity index detected in the HCHG group.

Consistent with the high opacity index, the HCHG group showed a high prevalence of corneal clouding (80.3 ± 5.8%) in zebrafish eyes. In contrast, corneal clouding was significantly reduced in the PCO (29.2 ± 4.2%, *p* < 0.001), ATV (51.3 ± 3.5%, *p* < 0.01), and ATV + PCO (36.4 ± 5.2%, *p* < 0.001) supplemented groups compared with the HCHG group (Supplementary Figure S1). 

Consistent with the corneal clouding outcomes, a notable change in characteristic iris color from silver bluish green to pale yellow silver gray appeared in the eyes of zebrafish consuming HCHG (Figure 4C). Compared with the ND (control) group, the HCHG group showed a 65.3% higher (*p* < 0.001) yellow color intensity. The zebrafish in the HCHG co-supplemented with PCO and ATV + PCO groups substantially protected the characteristic iris color (silver bluish green), with a significant 53.2% (*p* < 0.001) and 45.4% (*p* < 0.001) reduction in yellow color intensity compared to the yellow color intensity of the iris detected in the HCHG group. 

The eye morphological analysis suggests the beneficial effects of PCO and ATV + PCO on the prevention of HCHG-induced corneal clouding and the unusual color change in the iris.

### 2.5. Histological Analysis of Retina

H&E staining of the retina from the ND (control) group showed uniform cell density and compact cellular arrangement in the inner nuclear layer (INL) of the retina (Figure 5A,B). Also, a continuous and well-organized outer plexiform layer (OPL) and a broad, densely packed outer nuclear layer (ONL) appeared in the ND control group. In contrast, the HCHG-supplemented group showed a disorganized INL with void space, a relatively narrow ONL, and less distinction between OPL and ONL. Similarly, ATV co-supplementation failed to show any marked changes in the eye (retina) damage caused by exposure to HCHG. In contrast, PCO supplementation and ATV + PCO effectively minimized HCHG-induced changes in the INL, OPL, and ONL regions, supporting their efficacy in protecting the retina from HCHG-induced damage.

DHE and AO fluorescent staining suggest a significant 33.9-fold (*p* < 0.001) and 60.3-fold (*p* < 0.001) higher ROS and apoptosis in the HCHG-consumed group compared to the ND control group (Figure 5C,D,F,G). Supplementation of PCO and ATV + PCO effectively reduced HCHG-provoked ROS generation and apoptosis, as reflected by a significant 3.9-fold (*p* < 0.001) and 2.1-fold (*p* < 0.01) reduction in DHE fluorescence and 3.5-fold (*p* < 0.001) and 2.1-fold (*p* < 0.001) reduced AO fluorescence in the PCO- and ATV + PCO-supplemented groups, compared to the HCHG-supplemented group. Supplementation with ATV showed some reduction in HCHG-induced ROS and apoptosis; however, the difference was not statistically significant (*p* > 0.05).

IHC staining showed the highest myelin-specific fluorescence (7.4 ± 1.5 × 10^3^ AU) in the retina of the ND control group, which was 5.2-fold (*p* < 0.01) higher than the myelin-specific fluorescence detected in the HCHG group (1.4 ± 0.1 × 10^3^ AU) (Figure 5E,H). PCO and ATV + PCO substantially improved HCHG-induced retinal demyelination. Compared with the HCHG group, the PCO- (5.4 ± 0.6 × 10^3^ AU) and ATV + PCO (3.5 ± 0.9 × 10^3^ AU)-supplemented groups showed substantially higher myelin-specific fluorescence. 

The eye outcomes underscore the impact of PCO and the ATV + PCO in preventing HCHG-induced corneal clouding and morphological/pathological changes in zebrafish retinas.

### 2.6. Plasma Microinjection into Zebrafish Embryos

The functionality of plasma from different groups was assessed based on its ability to protect zebrafish embryos against CML-induced stress. As shown in Figure 6A, the Kaplan–Meier survival probability analysis revealed a substantial difference in embryo survival among the plasma-treated groups (log-rank *χ*^2^ = 225.6, *p* < 0.001). Following CML exposure, embryo survival probability began to decline after 5 h post-treatment (0.56), then further decreased to 0.21 at 24 h and to 0.12 after 72 h post-treatment. In comparison, the phosphate-buffered saline (PBS)-treated control group showed the highest survival probability of 0.83 at 72 h post-treatment (Figure 6A,B). Treatment with plasma obtained from the different experimental groups substantially improved the survival probability of CML-exposed embryos. Among the groups, plasma from the ND group showed the highest survival probability (0.72), whereas plasma from the HCHG group showed the lowest survival probability (0.49). Notably, plasma from HCHG co-supplemented with PCO, ATV, and ATV + PCO resulted in higher embryo survival probabilities of 0.66, 0.57, and 0.59, respectively, compared with the HCHG group (0.49), indicating differences in plasma functionality between the groups.

In response to CML, high ROS generation detected by DHE fluorescent staining was observed in the CML-injected group, which was significantly 7.1-fold (*p* < 0.001) higher than the ROS levels detected in the PBS (control) group (Figure 6C,F). CML-induced ROS generation was significantly prevented by plasma treatment from the ND, PCO, and ATV + PCO groups, as reflected by 6.2-fold (*p* < 0.001), 2.7-fold (*p* < 0.001), and 1.8-fold (*p* < 0.001) lower DHE fluorescence intensities compared to the DHE fluorescence intensity detected in only CML-injected embryos. A non-significant reduction in CML-induced ROS generation groups was observed following treatment with plasma from HCHG and ATV groups.

A great extent of apoptosis was observed in the CML-injected group, which was notably 8.5-fold (*p* < 0.001) higher than the extent of apoptosis observed in the PBS (control) group (Figure 6D,F). Treatment with plasma from the HCHG and ATV group did not significantly reduce CML-induced apoptosis. However, plasma from HCHG co-supplemented with ND, PCO, and ATV + PCO significantly reduced apoptosis by 6.1-fold (*p* < 0.001), 2.3-fold (*p* < 0.001), and 2.1-fold (*p* < 0.001), respectively. The CML-treated group also showed the lowest somite count (5.0 ± 0.6), which increased substantially to 28.6 ± 0.9, 16.3 ± 0.9, 27.7 ± 0.9, 20.0 ± 1.0, and 23.7 ± 0.7 after treatment with plasma from the ND, HCHG, PCO, ATV, and ATV + PCO groups, respectively (Figure 6E,G).

### 2.7. Developmental Defects and Swimming Activity

Outcomes from the 72 and 144 h post-treatments suggest developmental and swimming defects in many embryos (~45%) treated with CML (Figure 6B and Figure 7). Most of the embryos appeared with an uninflated swimming bladder, altered stationary posture, and severely compromised swimming ability. In contrast, the PBS (control)-treated group showed normal developmental morphology and swimming ability. Exposure to plasma from the HCHG-, PCO-, ATV-, and ATV + PCO-supplemented groups substantially protected against CML-impaired developmental defects and swimming abnormalities. However, about ~20% of embryos in the HCHG plasma-treated group appeared with some developmental deformities related to tail fin curvature, yolk sac edema, pericardial edema, stunted growth, and swimming abnormalities. Likewise, a substantial number of embryos (~11%) in the plasma from the HCHG co-supplemented with ATV group appeared with developmental deformities and impaired swimming movements. In contrast, most embryos treated with plasma from the HCHG co-supplemented with PCO and ATV + PCO groups showed normal embryo developmental morphology and swimming activity.

## 3. Discussion

Excessive consumption of HC and HG is known to cause several detrimental effects and serve as an ideal dietary model to induce metabolic stress that resembles the pathophysiology of human metabolic disorders. Specifically, the effect of galactose has been well documented to impair brain function and cause cognitive disorders [3,39]. Moreover, studies have documented that the consumption of a high amount of galactose (40%) crosses the placenta and impairs brain function in the developing fetus [40]. Galactose has been shown to impair antioxidant defenses, including superoxide dismutase (SOD), catalase, and glutathione peroxidase GSH-Px [41], and increases ROS generation and oxidative stress [42], potentially contributing to brain impairment and cognitive disorders [42,43,44,45]. Consistently, a 22-week consumption of HCHG leads to brain damage and severe cognitive alteration in adult zebrafish, which was substantially prevented by the intake of PCO and ATV + PCO. The positive effects of PCO and ATV + PCO on brain damage resulted from their ability to inhibit ROS generation and oxidative stress, as reflected by the current DHE fluorescent staining. Moreover, the earlier 22-week feeding study in zebrafish highlights a low level of the oxidative marker malondialdehyde (MDA) and a higher level of antioxidants such as sulfhydryl content, ferric ion reduction ability (FRA), and paraoxonase (PON) activity in hyperlipidemic, hyperglycemic zebrafish consuming PCO and ATV + PCO [33]. The effects of PCO and ATV + PCO in preventing brain damage may be associated with their ability to work as antioxidants that curtail ROS generation and oxidative stress. Compared with PCO and ATV + PCO, ATV alone exhibited a weaker brain-protective effect, which may be associated with its limited ability to modulate HCHG-induced oxidative stress-related parameters, as observed in the same cohorts of zebrafish in our preceding study [33]. Additionally, galactose exposure has been reported to induce chronic oxidative stress and alter gut microbiota [46], which may adversely affect brain health by disrupting the gut–brain axis [47]. Additionally, policosanol has been reported to positively modulate gut microbiota [48] and influence the stress response by altering microbial populations and prompting the production of beneficial microbial metabolites [49]. These events further support brain health by contributing to the restoration and maintenance of gut–brain homeostasis. Furthermore, in our previous study, we demonstrated that PCO and ATV + PCO effectively attenuated liver and kidney damage induced by HCHG [32]. Protecting hepatic and renal health can further support brain function via a well-established association between the liver and brain [47] and the kidney–brain axis [50].

Co-supplementation with PCO and ATV + PCO substantially inhibited high ROS generation, apoptosis, and senescent cells in response to HCHG. The substantial antioxidant nature of PCO and ATV + PCO has been documented [32] and may protect the brain from oxidative injury caused by an intake of HCHG. In addition, lower apoptosis and senescent-positive cells in the brain of PCO and ATV + PCO can be linked to lower ROS, as oxidative stress is a key contributor to senescence [51,52] and apoptosis [53]. This notion is supported by earlier reports deciphering the effect of antioxidants in preventing brain damage, senescence, apoptosis, and cognitive deficits induced by exposure to galactose [39]. Adverse effects of galactose on the myelin sheath have been reported [3]; the myelin sheath is an important multicellular membrane of the central nervous system (CNS) and is linked to cognitive processes and brain health [54]. Co-supplementation with PCO and ATV + PCO notably prevented substantial demyelination in response to HCHG. The beneficial roles of PCO and ATV + PCO are attributed to their notable effects on inhibition of ROS generation in the brain, an important factor in provoking demyelination [55], cognitive impairment, and brain aging [56].

Accumulation of high galactose in the brain leads to the formation of AGEs [42], which leads to cognitive impairment, brain damage, and aging [42,57]. Herein, swimming behavior and memory-related performance in zebrafish were assessed using the novel tank and passive avoidance tests, which are widely used to evaluate cognitive function in zebrafish [58]. The results show that HCHG intake causes substantial changes in swimming ability, indicated by geotaxis behavior (signs of anxiety-like behavior), and memory impairment, reflected by confused movement in response to electric pulses/passive avoidance tests. PCO and ATV + PCO intake provide a beneficial effect against HCHG-induced brain damage, primarily by inhibiting ROS generation and oxidative stress in the brain, which are considered important factors in brain damage and cognitive alterations [59]. We speculated that the antioxidant effects of PCO and ATV + PCO are key factors for improved brain health and reduced cognitive changes provoked by HCHG intake. In addition to its antioxidant activity, PCO’s strong antiglycation ability [16] may help to prevent HCHG-induced cognitive impairment by limiting AGE formation. These findings align with earlier reports documenting the ameliorating effect of policosanol on cognitive impairment [60]. Consistently, a study conducted in rodent models suggests a modulatory effect of policosanol on antioxidants and oxidative variables, thereby preventing Alzheimer’s related cognitive decline [61] and memory improvement evaluated by the passive avoidance test [62]. Combined brain and behavioral outcomes highlight the beneficial effects of PCO and ATV + PCO on brain health, memory, and cognitive changes in zebrafish. Additionally, the direct effects of ATV and PCO in attenuating HCHG-induced dyslipidemia have been recognized [33], which can substantially contribute to the prevention of brain health and cognitive function. This notion is consistent with previous findings showing a strong association between elevated cholesterol levels and impaired brain health and cognitive function [63]. Similarly, another study reported that lipid-lowering agents were associated with improvements in cognitive function among patients with dyslipidemia [64], underscoring the beneficial role of lipid-lowering therapy in preventing a decline in brain health.

HCHG have been documented to adversely affect the eyes, including the retina and the progression of cataracts. Lipid metabolism disorders characterized by dyslipidemia are closely related to the progression and development of ocular disease [65]. A preventive role of the substance in regulating lipid metabolism has been documented for its beneficial effects in ocular disease [65]. An evident effect of PCO and ATV on HCHG-induced dyslipidemia has been observed in the same cohorts of zebrafish in our preceding study [33]; therefore, they may prevent HCHG-induced eye damage.

Ocular sections (retina and cornea) are vulnerable to oxidative stress under conditions such as hypercholesterolemia and hyperglycemia [6,66]. Herein, in response to HCHG-induced severe oxidative stress and apoptosis in the ocular section of the retina, corneal opacity was significantly mitigated by co-supplementation with PCO and ATV + PCO. We assume that PCO’s antioxidant ability explains eye protection in the PCO- and ATV + PCO-supplemented groups against HCHG-induced oxidative damage, consistent with findings documenting the beneficial effects of antioxidants in protecting the eyes [66]. Similarly, cacao nuts have been reported to attenuate lens opacity in diabetic rats [67], potentially through their antioxidant ability and reduction in oxidative stress, highlighting the possible contribution of antioxidants to ocular protection. In addition to antioxidant properties, PCO and ATV + PCO have been shown to enhance HDL-C levels in hyperlipidemic and hyperglycemic zebrafish [32,33], which may also contribute substantially to eye protection. This aligns with earlier findings linking corneal opacity and HDL-C levels [68,69].

CML is a common AGE and is known to induce oxidative stress and inflammation [70]. Herein, embryos exposed to CML displayed higher oxidative stress, developmental defects, and death. Exposure to plasma from different groups, particularly the PCO- and ATV + PCO-supplemented groups, was associated with greater protection against CML-induced oxidative damage and developmental deformities. The findings suggest that plasma from PCO and ATV + PCO has higher functionality in mitigating the oxidative challenge, which may be associated with their superior antioxidant potential. The outcomes are in accordance with the earlier findings, where the same cohorts of zebrafish (fed PCO and ATV + PCO) resulted in lower oxidative species and higher antioxidant variables like FRA and PON activities in plasma [33]. Furthermore, the superior antioxidant ability supports the earlier findings where improved brain and eye health and cognitive improvement were observed in response to PCO- and ATV + PCO-supplemented zebrafish [32].

Limitations and prospective studies: This study demonstrates the beneficial effects of PCO and ATV + PCO on oxidative stress; however, the molecular mechanisms at the gene and protein levels, particularly the involvement of Nrf2/HO-1/NQO1 signaling, were not investigated, which remains a limitation of the present study. Also, the specific plasma factors or antioxidant components responsible for embryo-protective effects were not identified. These limitations will be addressed in future studies. In addition, the present study compared PCO and ATV at similar doses rather than at pharmacologically equivalent doses. Future investigations incorporating pharmacokinetic and dose–response analyses of PCO and ATV will be conducted to establish their pharmacologically comparable doses. Furthermore, the present investigation was conducted exclusively in a zebrafish model. As zebrafish are cold-blooded, their physiological and metabolic processes differ from those of warm-blooded humans. Therefore, these findings should be further validated in different warm-blooded animals. Due to these variations, the findings of the study should be carefully interpreted in the context of applicability in humans.

## 4. Materials and Methods

### 4.1. Materials

Atorvastatin (PHR1422-1G) was procured from Sigma-Aldrich (St. Louis, MO, USA). Policosanol (batch no. 310030324, 90.37% purity), extracted from Cuban sugarcane wax, was a mixture of eight long-chain aliphatic alcohols (LCAAs), namely tetracosanol (C_24_, 0.05%), hexacosanol (C_26_, 3.34%), heptacosanol (C_27_, 0.82%), octacosanol (C_28_, 60.02%), nonacosanol (C_29_, 0.55%), triacontanol (C_30_, 14.96%), dotriacontanol (C_32_, 8.24%), and tetratriacontanol (C_34_, 2.39%). Policosanol was provided by Raydel Pty Ltd., Thornleigh, NSW, Australia. The detailed specifications of policosanols are presented in Supplementary Table S1. 

The myelin-specific polyclonal primary antibody (0.7 mg/mL) was in-house produced in rabbits and was kindly provided by Prof. Cheol-Hee Kim, Laboratory of Developmental Genetics, Department of Biology, Chungnam National University, Daejeon 34134, Republic of Korea. A fluorescently tagged secondary antibody, goat anti-rabbit IgG (Alexa Fluor™ 405, A-31556), was purchased from Invitrogen (Waltham, MA, USA). All other chemicals used were of analytical grade and used as supplied unless otherwise stated.

### 4.2. Zebrafish Rearing

A 16-week-old wild-type AB strain of zebrafish (*D. rerio*) was housed in a water tank connected to a constant water supply. The water temperature was maintained at 28 °C, and the light and dark photo periods for zebrafish maintenance were autoregulated at 14 h and 10 h, respectively. The water had a chlorine content of 0.18 mg/L and a turbidity of 16 nephelometric turbidity units (NTU), with 8 mg/L dissolved oxygen (DO) and pH 7.3. The water was free from pathogenic microbes and had total bacterial counts < 100 CFU. The water analysis certificate is provided as Supplementary Table S2. The zebrafish were reared following the standard guidelines of Animal Care and Use, as adopted by the Raydel Research Institute (RRI-24-001, approval date: 2 September 2024). Zebrafish were fed the normal diet (ND, Tetrabit GmbH, D49307, Melle, Germany) at 9 am and 6 pm daily. Zebrafish were kept under these conditions for one week to acclimatize to the environment prior to processing for further study.

### 4.3. Preparation of Different Diets and Feeding of Zebrafish in Different Groups

A HCHG diet was prepared by mixing 4% (*w*/*w*) cholesterol and 30% (*w*/*w*) galactose in the ND. The HCHG diet was separately enriched with 0.05% (*w*/*w*) policosanol (PCO), 0.05% (*w*/*w*) atorvastatin (ATV), and 0.05% (*w*/*w*) each of atorvastatin and policosanol, and named as HCHG + PCO, HCHG + ATV, and HCHG + ATV + PCO diets. Supplementary Method Section S1 describes the detailed procedures for preparing the different formulated diets.

Zebrafish (*n* = 210) were randomly assigned to five groups, with 42 zebrafish per group. Zebrafish (*n* = 42) in Group I were fed ND, while zebrafish in Group II were fed the HCHG diet. Zebrafish in Groups III, IV, and V were fed HCHG + PCO, HCHG + ATV, and HCHG + ATV + PCO formulated diets. Zebrafish from each group were randomly distributed into three tanks (3 tanks × 14 fish = 42). A specified diet (10 mg/zebrafish) was provided twice (at 9 am and 6 pm), equivalent to a cumulative diet of 280 mg/tank/day. Because each tank contained 14 fish, it received 140 mg of diet (10 mg/fish × 14 fish) in the morning (9 am), and a similar amount in the evening (6 pm). Consequently, each tank received a total of 280 mg of diet/day (equivalent to 20 mg of diet/fish/day). The zebrafish were maintained on their respective formulated diets for 22 weeks. 

The 0.05% ATV dose was selected based on an earlier study, in which zebrafish were fed an HCHG supplemented with 0.0–1.0% ATV [33]. After 4 weeks, total blood cholesterol (TC) and triglyceride (TG) levels decreased substantially with an increasing ATV concentration up to 0.05%, with no further significant reduction at higher concentrations. Therefore, we selected 0.05% as the lowest effective concentration for subsequent experiments. For comparative analysis, PCO was also used at the same concentration (0.05%). Notably, feeding HCHG to zebrafish induced hyperlipidemia and hyperglycemia, as indicated by elevated blood TC, TG, low-density lipoprotein cholesterol (LDL-C), and glucose, and decreased high-density lipoprotein cholesterol (HDL-C) [33].

Before proceeding with the main feeding experiment, the liking and disliking of zebrafish for the different diets were examined by the food consumption efficacy test using the following formula: [total amount of the given food (mg)-remaining amount (mg)/total amount of the food supplied (mg)] × 100. A 95–100% food consumption efficacy was observed within 15 min of dietary exposure, indicating that zebrafish have a similar preference for all the formulated diets.

### 4.4. Swimming Behavioral Analysis of Zebrafish

The behavioral analysis of zebrafish in different groups was conducted using an open field tank (25 cm × 6 cm × 20 cm) following video recordings of swimming behavior. The tank was divided into two equal portions by inserting a horizontal line in the middle of the tank, and the two portions were named the lower and upper halves, as depicted in Supplementary Figure S2. After 22 weeks of feeding, 10 zebrafish were randomly selected from a total of 42 fish/group, which were maintained in three tanks (3 tanks × 14 fish = 42) (as described in Section 4.3). Notably, at least 3 zebrafish were randomly selected from each tank for the swimming behavioral analysis to ensure representation from all the experimental tanks. The randomly selected zebrafish (*n* = 10) were placed in the open field tank and allowed to stay in the tank for 30 min to acclimatize to the environment. After acclimatization, zebrafish swimming behavior (1 min) was recorded using a digital camera (Canon EOS90D, Tokyo, Japan). The recorded video was analyzed using Any-Maze software (version 7.0, Kim and Friends, Seoul, Republic of Korea) to determine the swimming trajectory, swimming distance, and time spent in the lower and upper halves. For the swimming behavioral analysis, researchers were blinded to the allocated groups, which were coded with a non-revealing alphabetical code before the analysis.

### 4.5. Passive Avoidance Ability of Zebrafish

To assess the passive avoidance ability of zebrafish, a tank (30 cm × 20 cm × 24 cm) was divided into two vertical halves using a physical divider, with an opening at the bottom to allow free movement between the two compartments. One compartment was covered with a black sheet to create a dark area, while the other remained uncovered as a light area, as shown in Supplementary Figure S3. On the dark side, a 9 V DC power supply using stainless-steel electrodes positioned 14 cm apart, with a maximum current capacity of 0.5 A, was applied to induce mild electric shock/stress stimuli. Under these conditions, the nominal electric field strength was approximately 0.22 V/cm, and the actual voltage inside the tank was 3.2 V. After 22 weeks of feeding, zebrafish from different groups were individually placed on the dark side of the tank, and a video of their swimming ability (1 min) was recorded using a digital camera (Canon EOS90D, Tokyo, Japan). During the 1 min swimming period, two brief electric stimuli, each lasting for 2 s, were delivered at 10 s and 20 s. The swimming behavior of the zebrafish and their movement between light and dark compartments were recorded throughout the test. The swimming videos were processed using Any-Maze software (version 7.0, Kim and Friends, Seoul, Republic of Korea) to determine swimming trajectory, boundary crossing, and stay time on the light side. 

Notably, three zebrafish were randomly selected from each experimental group (with one fish selected from each of the three tanks per group, as described in Section 4.3). For the passive avoidance test, each zebrafish was tested individually, and the test was repeated three times for each fish. Thus, the boundary crossing and time spent in the light compartment for each experimental group were determined based on the performance of three zebrafish, with each fish tested three times (i.e., 3 zebrafish × 3 times).

### 4.6. Corneal Clouding

After 22 weeks of feeding, the eyes of the zebrafish were examined under a stereomicroscope (Motic SMZ 168, Hong Kong, China) equipped with a camera (Motic cam 2300, Hong Kong, China). Zebrafish were anesthetized before examination of the eyes by placing them in a 0.1% phenoxyethanol solution (for 2 min). Both eyes of the anesthetized zebrafish were examined under the microscope, and images were captured. The two eyes from individual zebrafish were not considered independent biological replicates. The individual fish was considered the experimental unit, and the opacity grade for a particular fish was determined based on the average opacity value of both eyes. Captured images were processed for the semi-quantitative evaluation of opacity index (OI) using the earlier described Cho and Ashu scale [71]. Corneal clouding was graded on a scale of 0 (no corneal clouding) to grade 5 (complete corneal clouding), and the opacity index was calculated using the equation:

$$\mathrm{Opacity index}\;(\mathrm{OI})=\frac{\sum \left[\mathrm{Grade}\times \mathrm{number of eyes in that stage}\right]}{\mathrm{Total number of eyes}}$$

### 4.7. Blood and Organ Collection

After 22 weeks of feeding, zebrafish across the groups were sacrificed by hypothermic shock, and blood was immediately obtained by creating an incision between the anal fin and the caudal fin [72]. Blood was collected with a micropipette and placed in a microcentrifuge tube. Notably, blood from all the zebrafish from each tank of the specified group (as mentioned in Section 4.3) was collected and pooled in a single tube and mixed with 1mM ethylenediaminetetraacetic acid (EDTA)–PBS in a 2:3 (*v*/*v*) ratio. Thus, three pooled blood samples were obtained for each treatment group, with each sample representing the blood from the zebrafish from the respective tank, as described in Section 4.3. The collected blood was centrifuged for 10 min at 6000 rpm to obtain plasma, which was stored at 4 °C for future use. Among all groups, blood from the zebrafish was collected after 12 h fasting.

Organs (brain and eyes) were collected by dissection under a stereo microscope (Motic SMZ 168; Hong Kong, China) and preserved in 10% formalin solution for subsequent histological analysis. For all histological analyses, zebrafish from three tanks in each experimental group were individually analyzed. For the histological and plasma functionality analyses, researchers were blinded to the allocated groups. Samples were coded with a non-revealing alphabetical code before analysis. 

### 4.8. Hematoxylin and Eosin and Cellular Senescence Staining

For the histological analysis, tissue (brain and eyes) was blocked in frozen-section solution (Surgipath FSC22 solution, Leica, Nussloch, Germany). The tissue embedded in frozen-section solution was kept in liquid nitrogen for solidification. The solidified tissue block was stored at −21 °C in a refrigerator for 24 h and then processed for tissue sectioning (7 μm) using a cryo-microtome (Leica, CM-1510S, Nussloch, Germany).

The histological changes in the brain and eyes (retina) were assessed by H&E staining following the earlier described method [73]. In brief, the tissue section (brain or retina) was covered with Ventana HE 600 hematoxylin solution (lot no. N11615, Roche, Tucson, AZ, USA). After 5 min incubation, the section was washed with water (1 min), 0.5% HCl, and again with water (~20 s). The section was treated with 0.05% ammonia water for ~10 s and then washed with water. The section was then treated with Ventana HE 600 eosin solution (lot no. H30444, Roche, Tucson, AZ, USA). After 1 min of staining, the section was washed with water, air-dried, and visualized under the microscope (Nikon, Tokyo, Japan).

Senescence in the brain was determined by senescent-associated β-galactosidase (SA-β-gal) staining. The tissue section (7 μm) was covered with ~500 μL of 0.1% 5-bromo-4-chloro-3-indolyl-β-D-galactopyranoside solution (X-gal). The section was incubated overnight at RT in a moist environment. Finally, the section was rinsed with PBS and analyzed under the microscope for detection of blue-stained senescent positive cells.

### 4.9. Immunohistochemical (IHC) Staining and Dihydroethidium (DHE) and Acridine Orange (AO) Fluorescent Staining

IHC staining was performed for detection of the myelin sheath in the brain and eye (retina), following the earlier described method [74]. In brief, tissue sections (7 μm thick) were washed with PBS and then blocked with 2.5% BSA for 30 min, followed by the addition of a 250×-diluted myelin-specific primary antibody (IgG) of rabbit origin. After overnight incubation (~14 h) at 4 °C, the section was washed with PBS (3 times), followed by the addition of goat anti-rabbit IgG (Alexa Fluor™ 405, A-31556, Invitrogen, Waltham, MA, USA), followed by 4 h of incubation in the dark. Finally, the section was washed (three times) with PBS and visualized under the fluorescence microscope at excitation (Ex, 401 nm) and emission (Em, 422 nm) wavelengths. For quantitative analysis, ImageJ software (https://image.net/ij, version 1.54u, assessed on 28 July 2026) was used to evaluate myelin-specific fluorescence.

For DHE and acridine orange AO fluorescent staining, a tissue section (7 μm thick) was covered with 200 μL of a solution of DHE (30 μM) and 5 mg/mL of AO. After 30 min of incubation at RT in the dark, the stained section was washed three times with PBS. The section was visualized under a fluorescence microscope at emission and excitation wavelengths of 505/535 nm and 585/615 nm for AO and DHE fluorescence, respectively. All images under the fluorescence microscope (across the groups) were captured at similar parameters concerning exposure time, intensity of light, and pinhole diameter. The captured fluorescent images were processed for the quantification of fluorescent intensity using ImageJ software (https://imagej.net/ij, version 1.54u, assessed on 28 July 2026). Researchers were blinded to the allocated groups, and samples were coded with a non-revealing alphabetical code prior to the analysis.

### 4.10. Microinjection of Plasma into Zebrafish Embryos

To produce embryos, male and female zebrafish were kept in the breeding tank and separated from each other using a physical divider. After ~16 h of separation, the divider was removed, and male and female zebrafish were allowed to mate uninterruptedly for 30 min in the dark. Afterwards, the produced embryos were collected, washed with water, and then transferred into sea salt solution (0.01% sea salt with 1 μg/mL methylene blue). The embryos (1.30 h post-fertilization) were assigned to seven different groups (*n* = 150/group). The embryos (*n* = 150/group) were divided into three independent replicates, with 50 embryos in each replicate (*n* = 3 × 50 = 150). Embryos in Group I and Group II were microinjected with 10 nL of PBS and 10 nL of PBS containing 500 ng of CML, respectively. Embryos in Groups III and IV were microinjected with 10 nL of plasma obtained from the ND and HCHG-fed zebrafish in the presence of 500 ng CML. Similarly, embryos in the V, VI, and VII groups received 10 nL of plasma from the PCO-, ATV-, and ATV + PCO-supplemented groups, respectively, in the presence of 500 ng CML. Notably, the three plasma replicates from the specified group, as described in Section 4.7, were tested separately in embryos. For each embryo group (*n* = 150), 50 embryos were tested with plasma obtained from zebrafish in tank I, while the remaining 50 embryos were treated with plasma obtained from zebrafish in tank II, and another 50 embryos were treated with plasma obtained from zebrafish in tank III, all belonging to the same specified group. The embryos in the different groups were monitored up to 72 h post-treatment to assess survivability, somite number, and developmental deformities, following the OECD (test no. 23) 2019 guidelines. Swimming ability of the embryos was assessed under a microscope equipped with a digital video recording camera at 144 h post-treatment.

### 4.11. Dihydroethidium (DHE) and Acridine Orange (AO) Fluorescent Staining of Embryos

Embryos (*n* = 10) from each group were randomly selected from three replicates (as described in Section 4.10), with at least three embryos selected from each replicate. The selected embryos were kept in a 24-well plate and stained with 200 μL of a solution of DHE (30 μM) and 5 mg/mL AO. After 30 min incubation at room temperature in the dark, the embryos were washed three times with PBS. Stained embryos were visualized under a fluorescence microscope at the emission and excitation wavelengths of 505/535 nm and 585/615 nm for AO and DHE fluorescence, respectively. All images under the fluorescence microscope (across the groups) were captured at similar parameters. The captured fluorescent images were processed for the quantification of fluorescent intensity using ImageJ software (https://imagej.net/ij, version 1.54u, assessed on 28 July 2026).

### 4.12. Statistical Analysis

All experiments were conducted at least in triplicate (*n* = 3), and the results are shown as the mean ± standard error of the mean (SEM). The statistical difference between the groups was established by one-way analysis of variance (ANOVA) using the SPSS platform (version 29, Chicago, IL, USA) at a 95% confidence level. Tukey’s post hoc analysis was conducted with statistical significance (*p* < 0.05) to establish the statistical differences between groups. Data normality was examined using the Kolmogorov–Smirnov test prior to performing ANOVA.

## 5. Conclusions

The findings demonstrate that PCO and ATV + PCO supplementation may provide substantial protection against HCHG diet-induced ocular and brain damage. PCO and ATV + PCO supplementation were associated with reduced corneal clouding and retinal damage, accompanied by attenuation of oxidative stress. In the brain, these treatments were associated with reduced ROS generation, cellular senescence, and demyelination, along with improvements in swimming behavior, anxiety-like responses, memory, and cognitive function. Collectively, the findings suggest that PCO and ATV + PCO exert protective effects against HCHG-induced impairments in the brain and eyes of zebrafish. Nevertheless, these findings need verification in other animal models and through preclinical studies to assess their utility and potential relevance to human health.

## Figures and Tables

**Figure 1:**
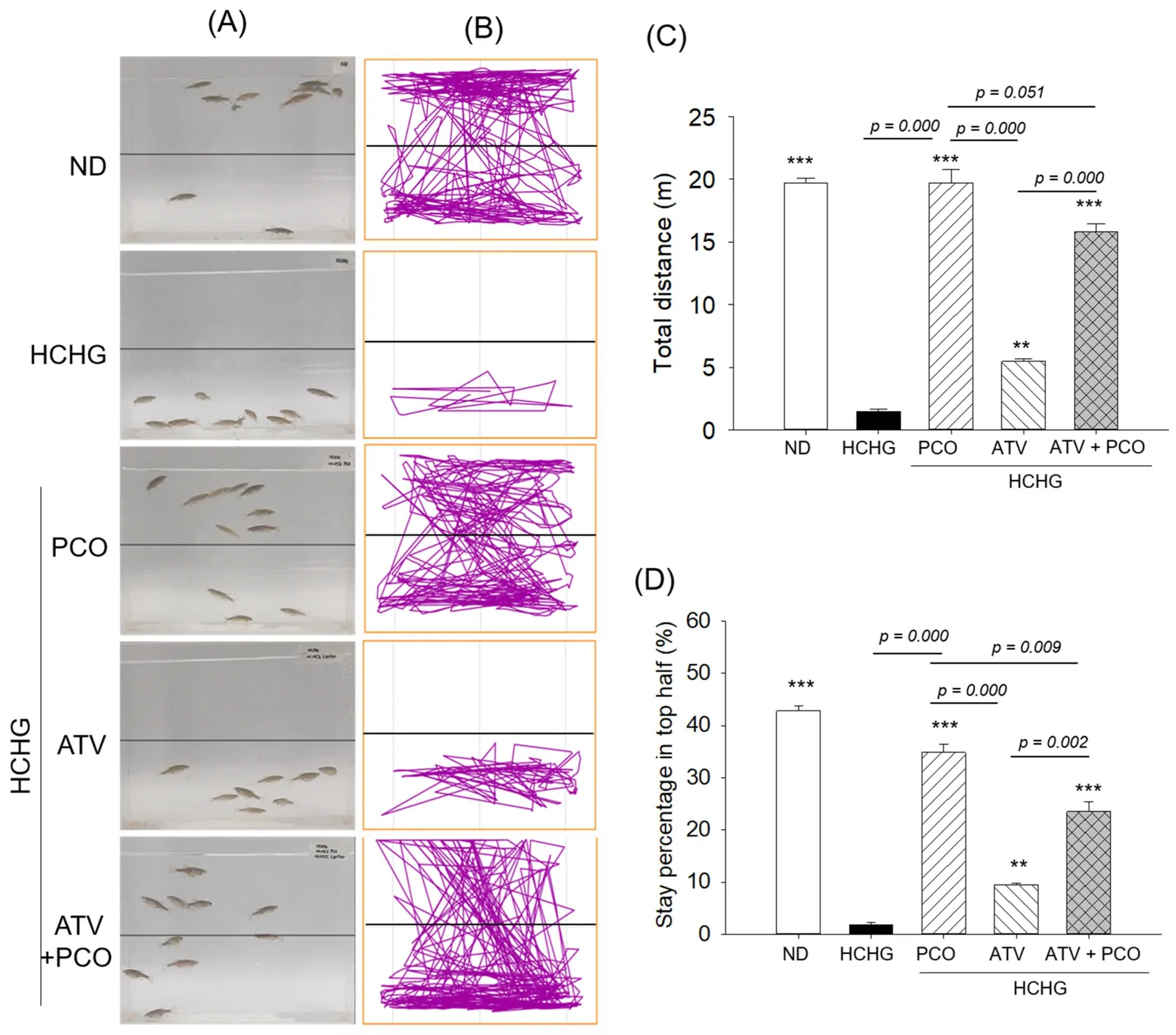
Zebrafish swimming ability (open field tank analysis) (*n* = 10) across the groups after 22 weeks of feeding the specialized diet. (**A**) Representative still images of zebrafish swimming positions in the tank. (**B**) Swimming trajectories of zebrafish, determined by tracking software (Any-Maze version 7.0, Kim and friends, Seoul, Republic of Korea). The black horizontal line in the image divides the tank into two halves (top and bottom). (**C**) Total swimming distance. (**D**) Average stay time of zebrafish in the top half of the tank. The swimming trajectory, total swimming distance, and stay time in the top half were obtained from the one-minute swimming experiment. Abbreviations: ND, normal diet; HCHG, high-cholesterol and high-galactose diet; PCO, policosanol; ATV, atorvastatin. ** (*p* < 0.01) and *** (*p* < 0.001) depict the statistical difference between the groups compared to the HCHG group. The *p*-value highlights statistical differences between the marked groups. All statistical differences were established using one-way ANOVA followed by Tukey’s multiple comparison test.

**Figure 2:**
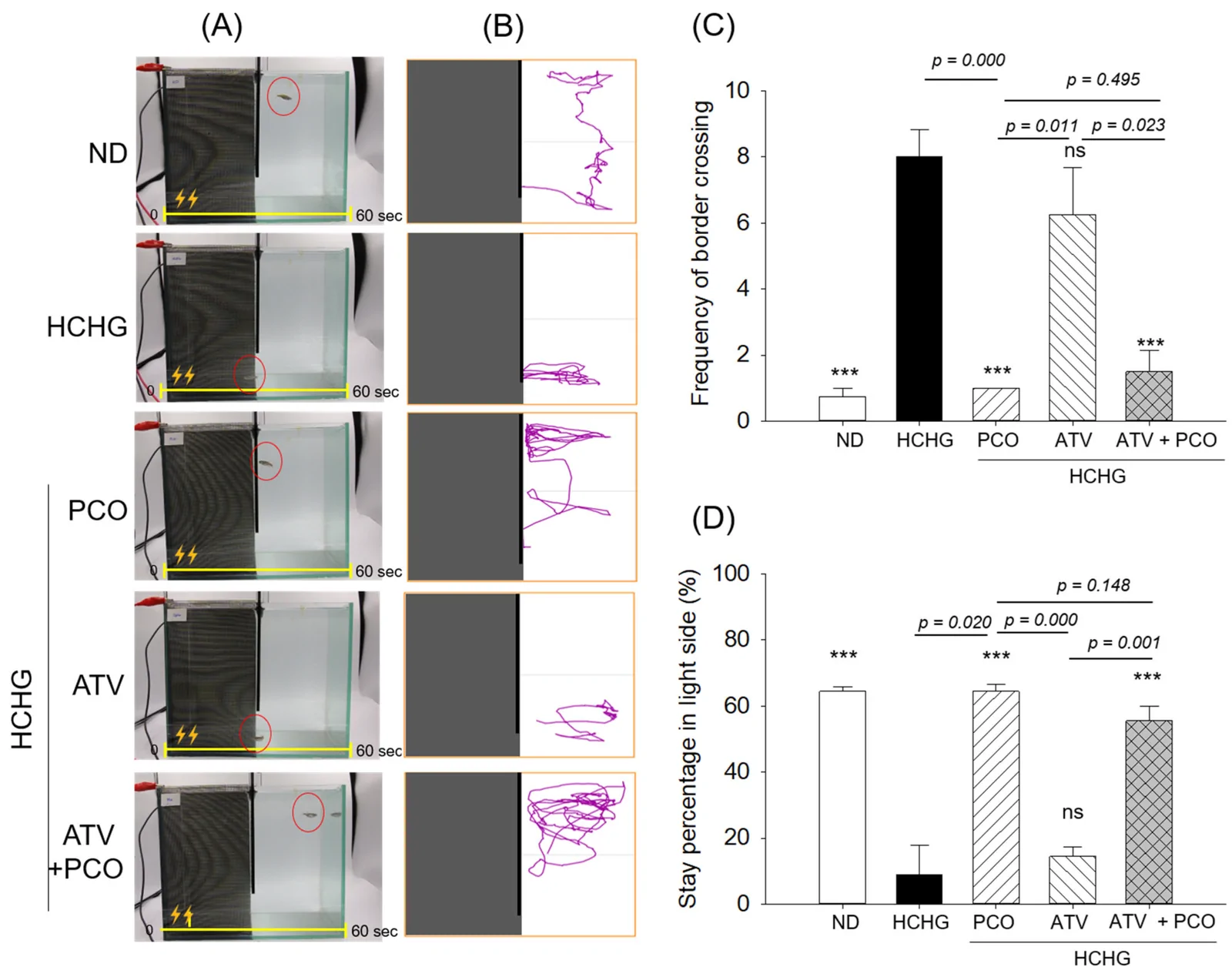
Passive avoidance ability and swimming behavior of zebrafish (*n* = 3/group) after 22 weeks of feeding designated diets. (**A**) Representative images of the tank divided into two vertical halves (dark and light). The light and dark halves are separated by a physical divider along the entire length but open at the bottom to allow free passage of zebrafish between the halves. (**B**) Swimming trajectories (in the light half) of zebrafish determined by tracking software (Any-Maze version 7.0, Kim and friends, Seoul, Republic of Korea). (**C**) Frequency of border crossing. (**D**) Average stay time of zebrafish on the light side of the tank. The swimming trajectory, border-crossing frequency, and stay percentage on the light side were obtained from a 1 min swimming experiment. Abbreviations: ND, normal diet; HCHG, high-cholesterol and high-galactose diet; PCO, policosanol; ATV, atorvastatin. *** (*p* < 0.001) depicts the statistical difference between the groups compared to the HCHG group; ns indicates non-significant differences. The *p*-value highlights statistical differences between the marked groups. All statistical differences were established using one-way ANOVA followed by Tukey’s multiple comparison test.

**Figure 3:**
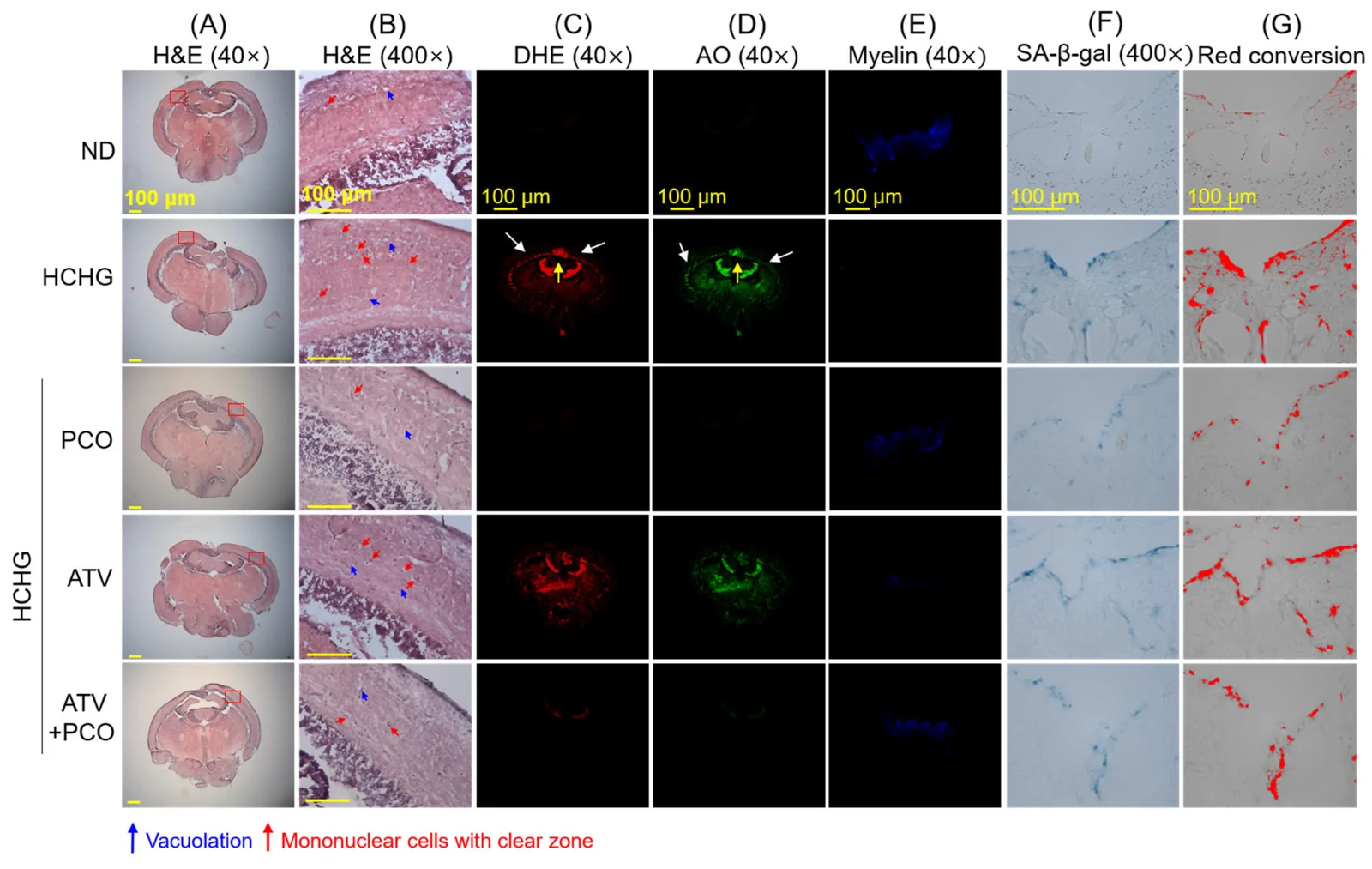

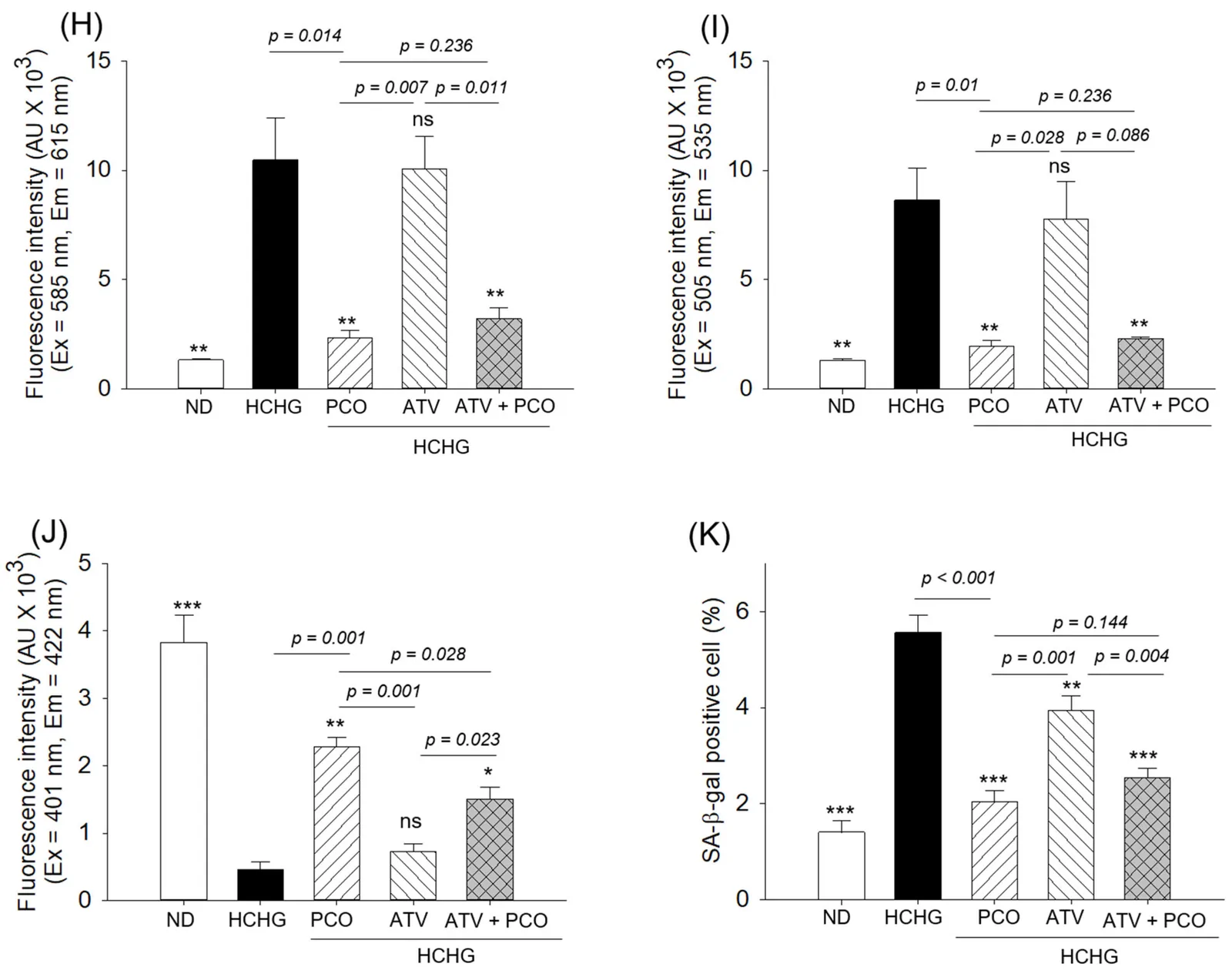
Zebrafish brain histology after 22 weeks of intake of the designated diets. (**A**) Hematoxylin and eosin staining of the brain at 40× magnification. (**B**) A magnified view (400×) of the H&E section covered under the red box (panel **A**). (**C**,**D**) Dihydroethidium (DHE) and acridine orange (AO) fluorescent staining. White arrows highlight the tectum opticum (TeO), while yellow arrows highlight the valvular cerebelli (Val) region. (**E**) Immunohistochemical (IHC) images of the myelin sheath in the brain. (**F**) Senescence-associated β-galactosidase (SA-β-gal) staining. (**G**) Red conversion of the SA-β-gal-stained blue area to improve visibility. The red conversion was performed using ImageJ software (https://imagej.net/ij, version 1.54u, assessed on 28 July 2026) at the blue color threshold value of 0–120. Quantification of (**H**) DHE, (**I**) AO, and (**J**) myelin fluorescent intensity. (**K**) Quantification of the SA-β-gal-stained area. For the quantitative analysis of fluorescence intensities and SA-β-gal-positive cells, three (*n* = 3) independent sections and five random fields per section were analyzed. Abbreviations: ND, normal diet; HCHG, high-cholesterol and high-galactose diet; PCO, policosanol; ATV, atorvastatin. * (*p* < 0.05), ** (*p* < 0.01) and *** (*p* < 0.001) depict the statistical differences between the groups compared to the HCHG group; ns indicates non-significant differences. The *p*-value highlights statistical differences between the marked groups. All statistical differences were established using one-way ANOVA followed by Tukey’s multiple comparison test.

**Figure 4:**
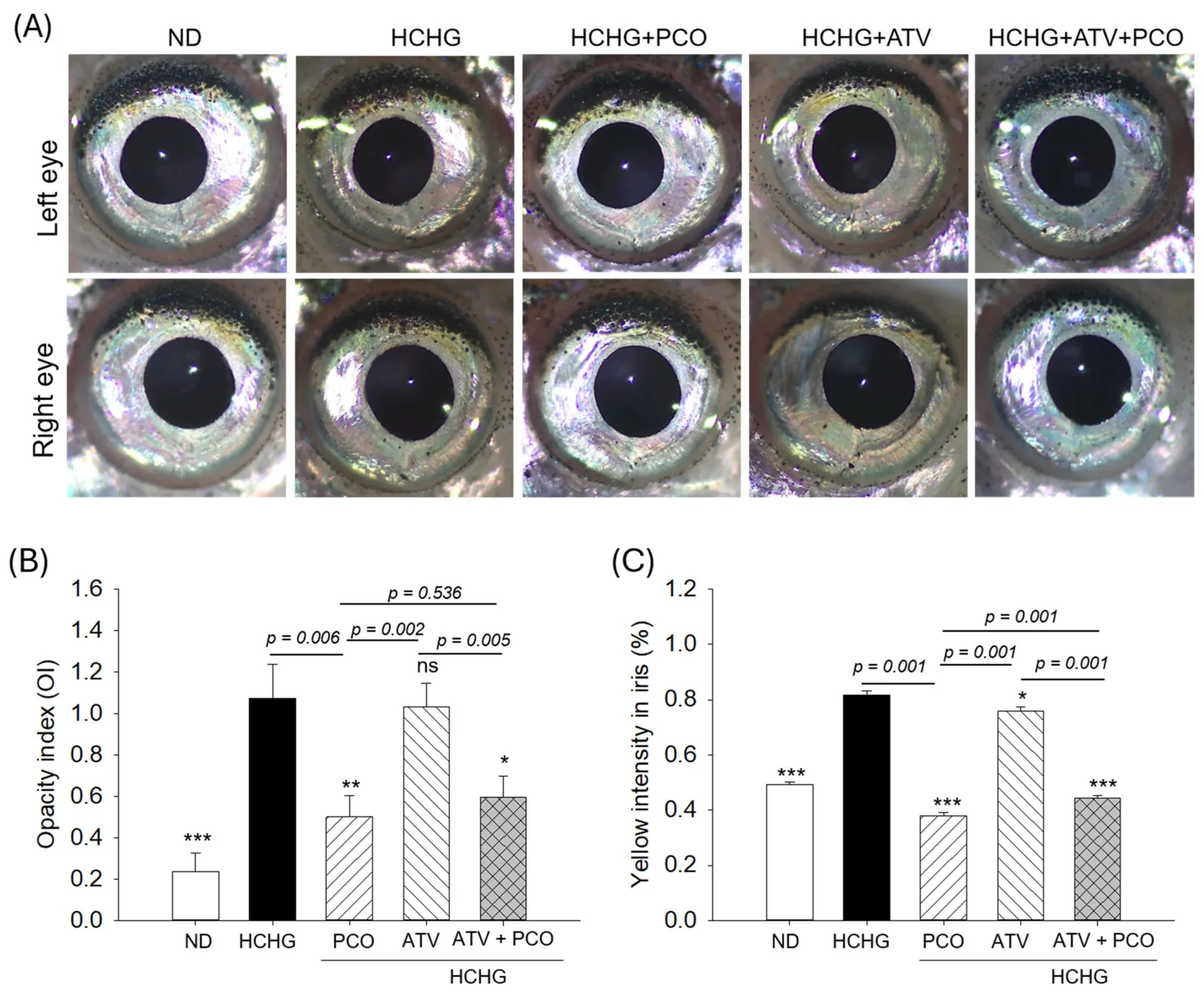
Morphology of zebrafish eyes after 22 weeks of consumption of specialized diets. (**A**) Representative right and left eye images of zebrafish. (**B**) Corneal opacity index and (**C**) intensity of yellow pigmentation in the iris. Abbreviations: ND, normal diet; HCHG, high-cholesterol and high-galactose diet; PCO, policosanol; ATV, atorvastatin. * (*p* < 0.05), ** (*p* < 0.01), and *** (*p* < 0.001) depict the statistical differences between the groups compared to the HCHG group; ns indicates non-significant differences. The *p*-value highlights statistical differences between the marked groups. All statistical differences were established using one-way ANOVA followed by Tukey’s multiple comparison test.

**Figure 5:**
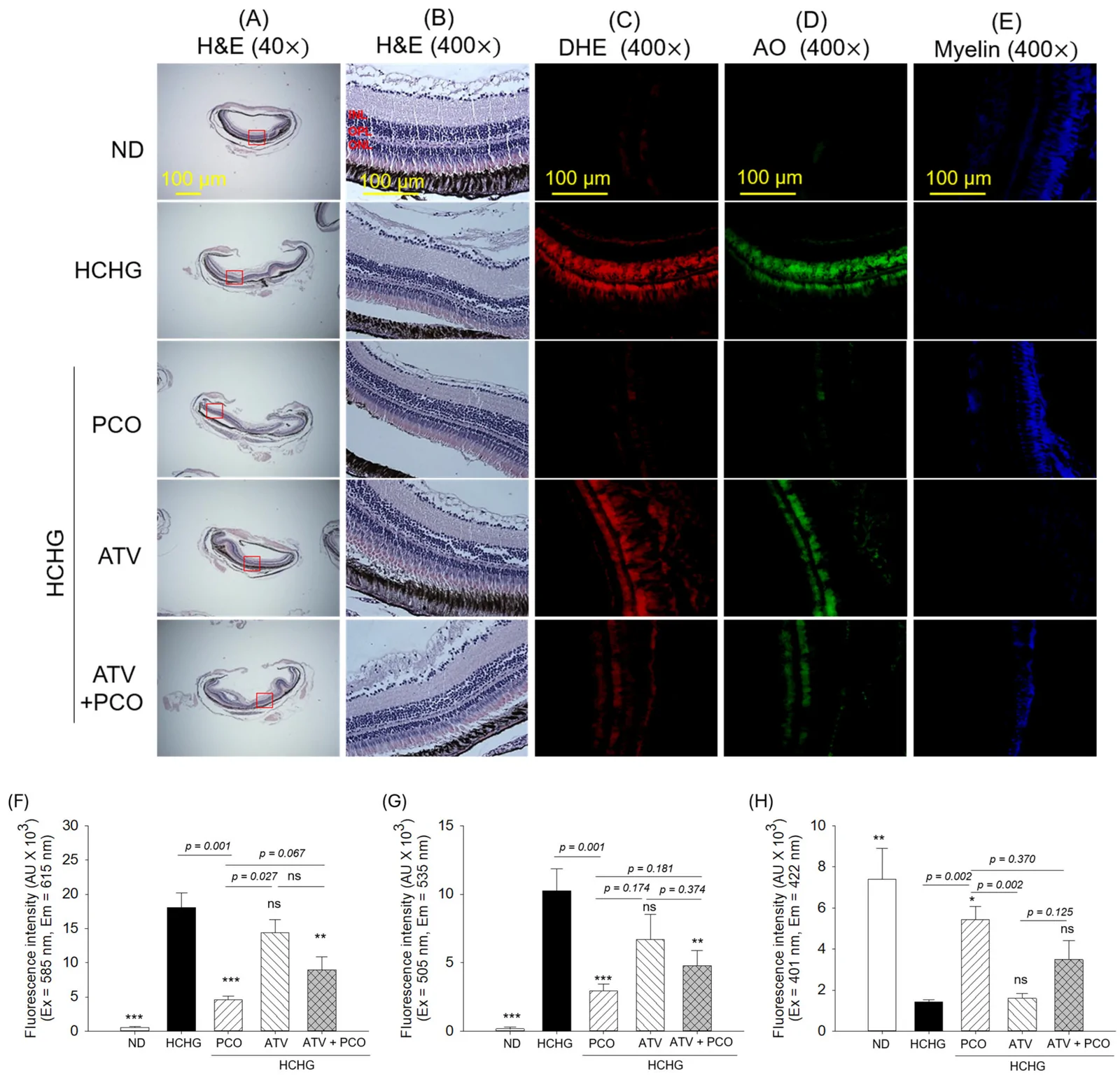
Zebrafish eye histology after 22 weeks of supplementation with the specialized diets. (**A**) Hematoxylin and eosin (H&E) staining of the eye (retina) at 40× magnification. (**B**) The magnified (400×) view of the H&E area covered within the red box of (panel **A**). INL, OPL, and ONL are abbreviations for the inner nuclear layer, outer plexiform layer, and outer nuclear layer, respectively. (**C**,**D**) Dihydroethidium (DHE) and acridine orange (AO) fluorescent staining, respectively. (**E**) Immunohistochemical (IHC) images of the myelin sheath. Quantification of fluorescent intensity corresponding to (**F**) DHE, (**G**) AO, and (**H**) myelin. For the quantitative analysis of fluorescence intensities, three (*n* = 3) independent sections and five random fields per section were analyzed. Abbreviations: ND, normal diet; HCHG, high-cholesterol and high-galactose diet; PCO, policosanol; ATV, atorvastatin. * (*p* < 0.05), ** (*p* < 0.01) and *** (*p* < 0.001) depict the statistical differences between the groups compared to the HCHG group; ns indicates non-significant differences. The *p*-value highlights statistical differences between the marked groups. All statistical differences were established using one-way ANOVA followed by Tukey’s multiple comparison test.

**Figure 6:**
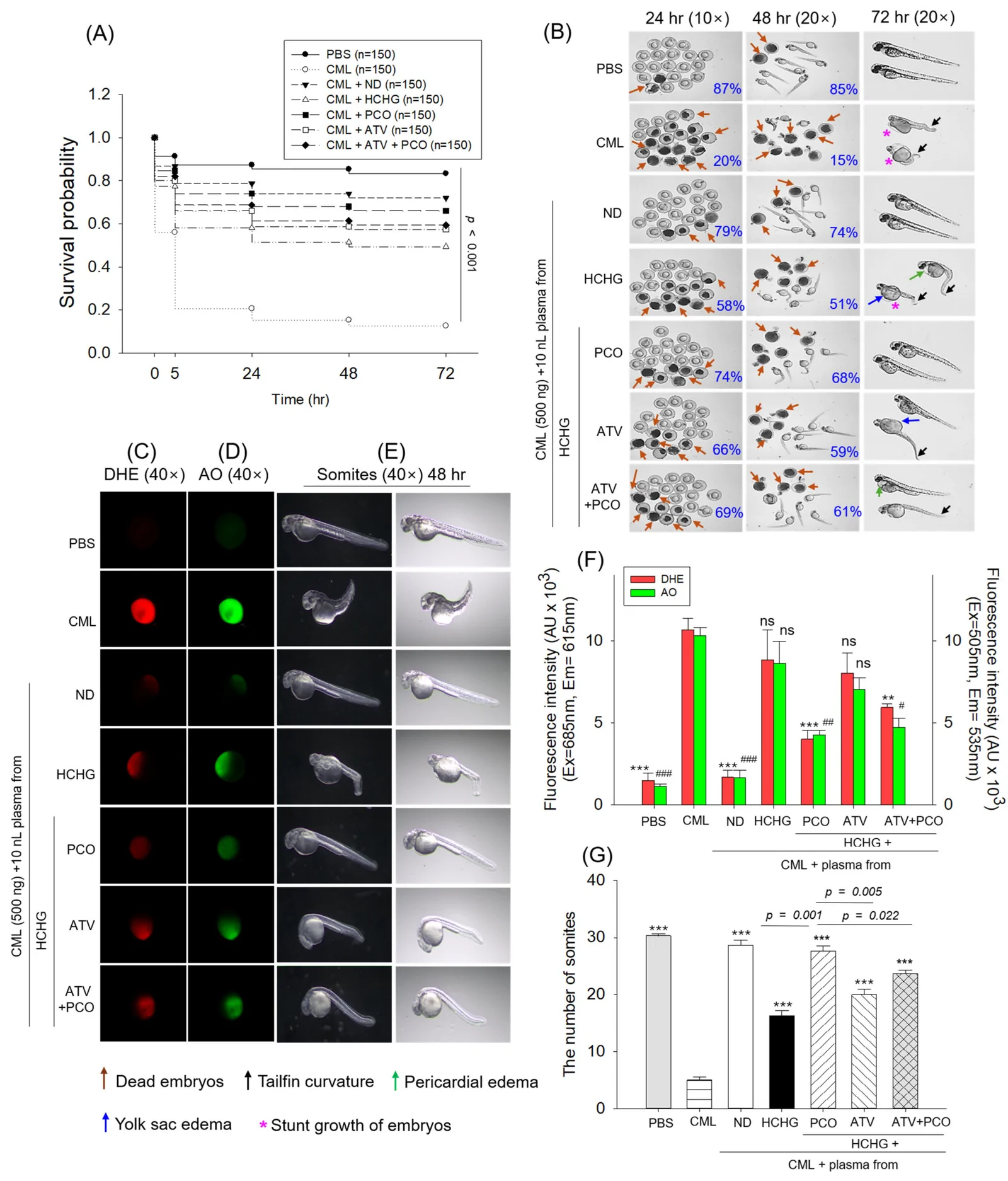
Effect of zebrafish plasma obtained from 22 weeks of feeding on specialized diets on carboxymethyllysine (CML)-induced toxicity in zebrafish embryos. (**A**) Kaplan–Meier survival curve of zebrafish embryos (*n* = 150) during 72 h post-treatment. (**B**) Pictorial view of zebrafish embryos at 24 h, 48 h, and 72 h post-treatment. (**C**,**D**) Dihydroethidium (DHE) (AO) and acridine orange fluorescent staining of the embryos (*n* = 10). (**E**) Images representing the somites in embryos. (**F**) Quantification of DHE and AO fluorescent intensities. (**G**) Quantification of the somite counts in the developing embryos. Abbreviations: PBS, phosphate-buffered saline; CML, carboxymethyllysine; ND, normal diet; HCHG, high-cholesterol and high-galactose diet; PCO, policosanol; ATV, atorvastatin. ** (*p* < 0.01) and *** (*p* < 0.001) depict the statistical differences between the groups compared to the CML-treated group (for DHE fluorescent intensity and somite counts), while ^#^ (*p* < 0.05), ^##^ (*p* < 0.01), and ^###^ (*p* < 0.001) depict the statistical differences (for AO fluorescent intensity) compared to the CML-treated group; ns indicates non-significant differences. The *p*-value highlights statistical differences between the marked groups. All statistical differences were established using one-way ANOVA followed by Tukey’s multiple comparison test.

**Figure 7:**
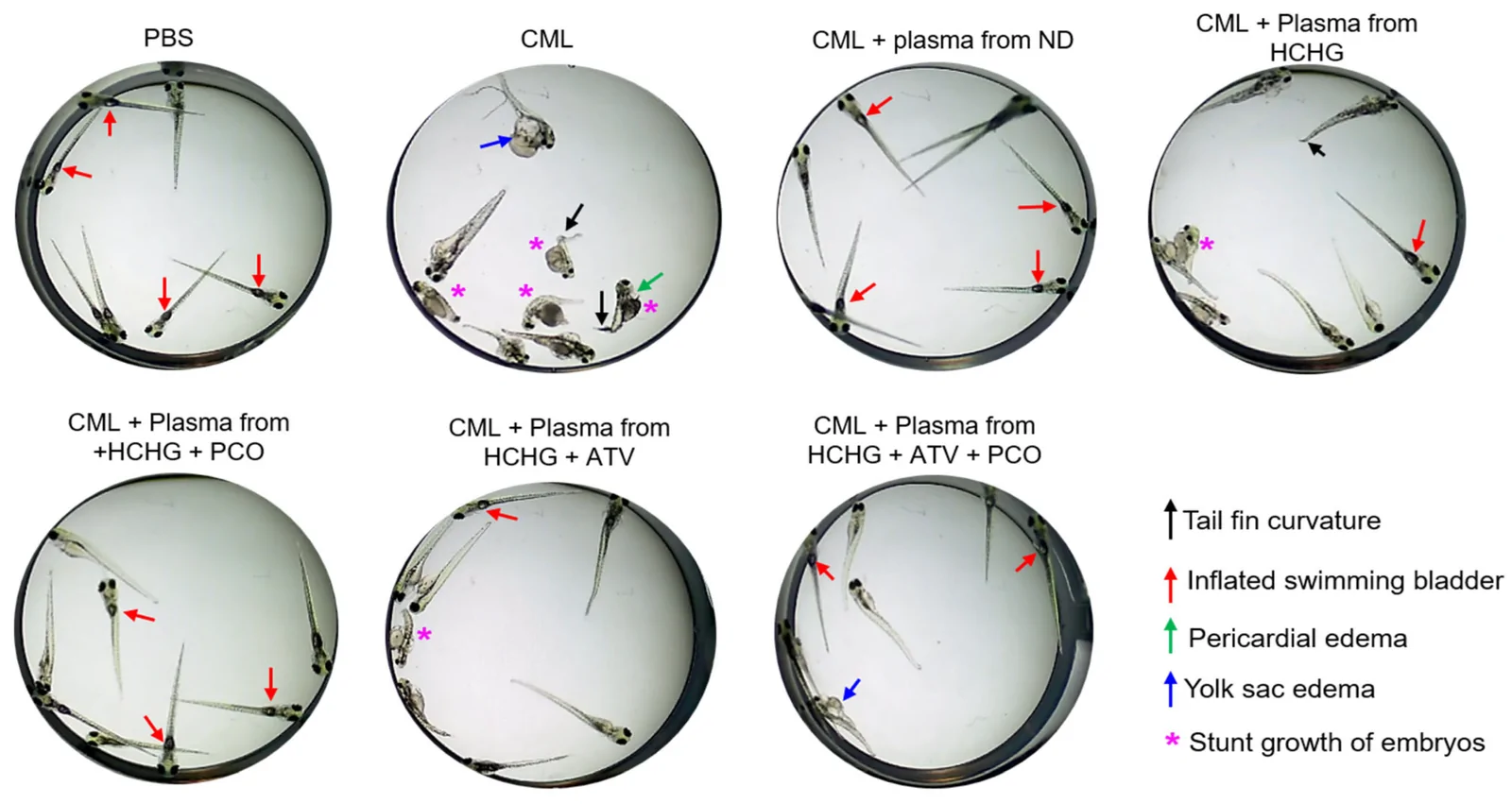
Images of zebrafish embryos (*n* = 8) at 144 h post-treatment with carboxymethyllysine (CML) in the presence of plasma from adult zebrafish consuming the specified diets for 22 weeks. Abbreviations: PBS, phosphate-buffered saline; CML, carboxymethyllysine; ND, normal diet; HCHG, high-cholesterol and high-galactose diet; PCO, policosanol; ATV, atorvastatin.

## Data Availability

The original contributions presented in this study are included in the article/Supplementary Materials. Further inquiries can be directed to the corresponding author.

## Supplementary Materials

The following supporting information can be downloaded at: https://www.mdpi.com/article/10.3390/ph19101622/s1, Supplementary Table S1: Certificate of analysis and the composition of the used policosanol. Supplementary Table S2: Certificate of water quality analysis. Supplementary Material Section S1: Method for the preparation of different diets. Supplementary Figure S1: A comparative corneal clouding incidences among the different groups of zebrafish post 22 weeks intake of the designated diets. Abbreviations: ND, normal diet; HCHG, high cholesterol-high galactose diet; PCO, policosanol; ATV, atorvastatin. ** (*p*< 0.01) and *** (*p* < 0.001) depict the statistical difference between the groups compared to the HCHG group. The *p*-value highlights statistical differences between the marked groups. All the statistical differences were established using one-way ANOVA followed by Tukey’s multiple comparison test. Supplementary Figure S2: Ray diagram of the open field water tank divided into two sectors (i.e., top half and lower half) using a dotted horizontal line. Supplementary Figure S3: Ray diagram of tank divided into two vertical halves (dark and light) used to perform passive avoidance test.
