# Supplementary material for: Co-Supplementation of Policosanol and Atorvastatin Attenuates High-Cholesterol and High-Galactose Diet-Induced Ocular and Brain Damage and Improves Cognitive Health in Zebrafish
Source: Pharmaceuticals (Basel). 2026 Oct 13;19(10):1622. doi: 10.3390/ph19101622 (PMC13708359; doi:10.3390/ph19101622)
Supplement: Supplementary file 1 [file pharmaceuticals-19-01622-s001.zip › pharmaceuticals-4582194-supplementary.pdf]

Supplementary materials

Supplementary Figure S1

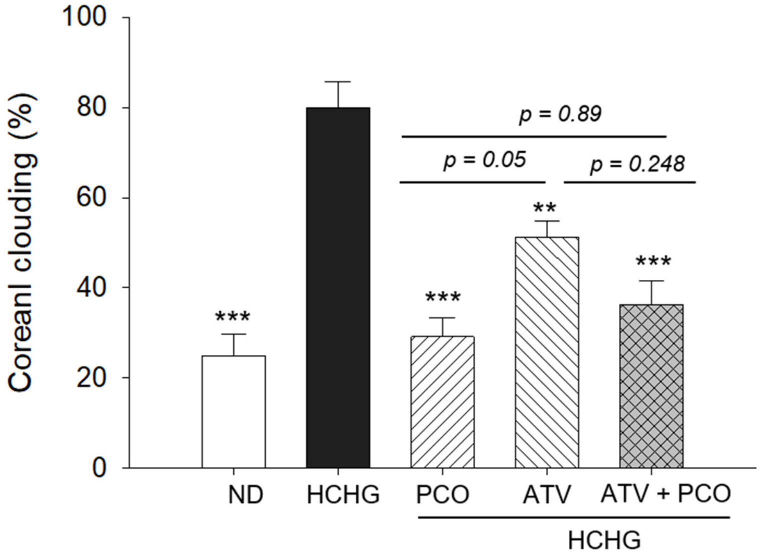

Supplementary Figure S1: A comparative corneal clouding incidences among the different groups of zebrafish post 22 weeks intake of the designated diets. Abbreviations: ND, normal diet; HCHG, high cholesterol-high galactose diet; PCO, policosanol; ATV, atorvastatin. ** ($p <$ 0.01) and *** ($p <$ 0.001) depict the statistical difference between the groups compared to the HCHG group. The *p*-value highlights statistical differences between the marked groups. All the statistical differences were established using one-way ANOVA followed by Tukey's multiple comparison test.

Supplementary Table S1

RAYDEL
Health Confidence

Raydel Australia Pty Ltd
Level 1, building 1, 9-15 Chilvers Rd.
Thornleigh NSW 2120 Australia
Tel +61 2 9480 1300
Fax +61 2 9480 1399
A.B.N. 45 054 555 903
www.raydel.com.au
info@raydel.com.au

Product name: Policosanol

Batch #: 310030324

Date of Manufacture: 12/03/2024

Parameter	Results	Approved Limits
Color	Complies	Off white to cream
**Identity and Purity***		
1-tetracosanol (C_{24})	0.05 %	0.00 – 2.0 %
1-hexacosanol (C_{26})	3.34 %	3.0 –10.0 %
1-heptacosanol (C_{27})	0.82 %	0.1 - 3.0 %
1-octacosanol (C_{28})	60.02 %	60.0 –70.0 %
1-nonacosanol (C_{29})	0.55 %	0.1- 2.0 %
1-triacontanol (C_{30})	14.96 %	10.0 – 15.0%
1-dotriacontanol (C_{32})	8.24 %	5.0 - 10.0 %
1-tetratriacontanol (C_{34})	2.39 %	0.1 – 5.0 %
**Total (Purity*)**	**90.37 %**	**≥ 90 %**
**Other quality specifications**		
**Melting temperature**	81.3-83.0 °C	78.0 – 83.0 °C
**Loss on drying**	0.54 %	≤ 1.0 %
**Residue of ignition**	0.72 %	≤ 0.85 %
**Heavy metals (Pb, Cd, Hg)**	<0.000015 %	≤ 0.001 %
**Sodium content**	86.34 ppm	≤ 100 ppm
**Potassium content**	2905.41 ppm	≤ 4500 ppm
**Residual solvents**		
**Acetone**	≤ 0.03	≤ 0.03 g/kg
**Hexane**	≤ 0.005	≤ 0.005 g/kg
**Microbiological content**		
**Total Aerobic Microbial Count**	≤10	$\leq 10^3$ per g
**Yeast and mould**	≤10	$\leq 10^2$ per g
**Enterobacteria or Coliform count**	≤10	$\leq 10^2$ per g
***Staphylococcus aureus, Pseudomonas aeruginosa, Escherichia* coli, Candida albicans**	Absent	Absent in 1 g
Salmonella sp	Absent	Absent in 10 g
Observations:		
**References:**	* Manufacturer GC validated method, purity expressed as the total of high molecular weight alcohols	

Note about storage conditions: No special storage conditions are required. The substance has a shelf life of 5 years stored under ambient conditions of climatic Zones IV or II, as demonstrated in stability studies performed according to ICH guidelines.

Approved (_X_) Released (_X_) Rejected (___)

This COA is it reproduced from supplier's COA

Table S1: Certificate of analysis and the composition of the policosanol used.

Supplementary Table S2

Original certificate of water quality

분석과학을 선도하는 기업
KIRIM (주)기림생명과학원
KiRim Institute of Life Science Co.,Ltd.

받 음: 대구 동구 동내로 76 (동내동)
한국메디벤처센터 지하2층 관리실 귀하
41061

수질검사성적서

수신: 한국메디벤처센터
제목: 먹는물(저수조수) 수질검사성적서 발급

발 급 일: 2025년 5월 2일
발급번호: KWCT3379961
발 신: (주)기림생명과학원

「먹는물 수질기준 및 검사 등에 관한 규칙」 제3조제2항에 따라 다음과 같이 먹는물 수질검사성적서를 발급합니다. 이 성적서는 의뢰인이 제시한 시료에 대한 결과이며 검사목적 이외에는 사용할 수 없습니다.

1. 시료 내용

접수번호	12506438	접 수 일	2025년 4월 28일
의 뢰 인	한국메디벤처센터		
시 료 명	저수조수		
검사목적	수도법(저수조수 수질검사)		
채수장소	대구 동구 동내로 76 (동내동)		
시료채취	기술인력채취 (주)기림생명과학원 성기현 채수일시 : 2025.04.28		
비 고	판정은 수도법 시행규칙 제22조4의⑤항 및 ⑦항에 따른 먹는물 수질기준 및 검사등에 관한 규칙[별표1]에 의거합니다.		

2. 수질검사 결과

검사항목	기준	검사결과
탁도	0.5 NTU 이하	0.16
수소이온농도	5.8 ~ 8.5	7.3
잔류염소	0.1 ㎎/L이상 4.0 ㎎/L이하	0.18
일반세균	100 CFU/mL이하	0
총대장균군	불검출/100mL	불검출
분원성대장균군	불검출/100mL	불검출
종합결과	적합	

• 탁도(등급외 성능),잔류염소(1등급)는 간이측정기를 사용하여 측정함.

(주)기림생명과학원 대표이사

- 환경과 생명을 소중히 하는 아름다운 기업 -

Certificate of water quality (translated in English)

Drinking Water Quality Test Report
Recipient: Korea Mediventure Center
B2 Management Office
76 Dongnae-ro, Dong-gu, Daegu (Dongnae-dong), 41061
Issued by: Kirim Life Science Co., Ltd.
Issue Date: May 2, 2025
Report Number: KWCT3379961
Subject: Drinking Water (Reservoir Water) Quality Test Report

Basis of Issuance
This report is issued in accordance with Article 3, Paragraph 2 of the *Regulations on Drinking Water Quality Standards and Testing*.
It presents the results of the sample provided by the client. The report must not be used for purposes other than water quality testing.

1. Sample Information
•**Receipt Number:** 12506438
•**Receipt Date:** April 28, 2025
•**Client:** Korea Mediventure Center
•**Sample Name:** Reservoir Water
•**Purpose of Test:** Waterworks Act (Reservoir Water Quality Test)
•**Sampling Location:** 76 Dongnae-ro, Dong-gu, Daegu (Dongnae-dong)
•**Collected by:** Technical staff (Seong Ki-hyun, Kirim Life Science Co., Ltd.)
•**Sampling Date/Time:** April 28, 2025
•**Remarks:** Evaluation conducted in accordance with Article 22, Paragraph 4 of the Enforcement Rules of the Waterworks Act and Annex 1 of the *Regulations on Drinking Water Quality Standards and Testing*.

2. Test Results

Test Item	Standard	Result
Turbidity	≤ 5 NTU	0.16
pH (Hydrogen Ion)	5.8 – 8.5	7.3
Residual Chlorine	0.1 – 4.0 mg/L	0.18
General Bacteria	≤ 100 CFU/mL	0
Total Coliform Group	Not detected / 100 mL	Not detected
Fecal Coliform Group	Not detected / 100 mL	Not detected

Comprehensive Result: Suitable (Compliant)

Note: Turbidity (non-graded performance) and residual chlorine (Grade 1) were measured using a simplified measuring device.

Certification
Issued by: **Kirim Life Science Co., Ltd.** Representative Director
- A beautiful company that values environment and life -

Table S2: Certificate of water quality analysis.

Section S1.

1. Preparation of different diets

For preparing the HCHG diet, 132 g of ND was supplemented with 8 g of cholesterol and thoroughly mixed with a spatula. Subsequently, chloroform (~ 100 mL) was added to the mixture (ND+cholesterol), and the mixture was mechanically agitated to ensure proper

distribution of cholesterol. Later, the chloroform was completely evaporated in a fume hood to obtain the high-cholesterol (HC) diet. The prepared HC diet was supplemented with 60 g of galactose, properly mixed with a spatula, and then distilled water (~ 300 mL) was added. The mixture was blended properly with a spatula and left for 4 hr at room temperature (RT). Finally, the mixture was re-agitated (~ 10 min) and processed for freeze-drying to evaporate water. The freeze-dried sample was named the HCHG diet containing 4% (*w*/*w*) cholesterol and 30% (*w*/*w*) galactose.

For the preparation of HCHG enriched with policosanol (PCO) and atorvastatin (ATV), 131.9 g of ND was supplemented with 8 g of cholesterol and mixed thoroughly using a spatula. Subsequently, chloroform (~ 100 mL) was added to the mixture, and the mixture was mechanically agitated to ensure proper distribution of cholesterol. The chloroform was then completely evaporated in a fume hood to obtain the HC diet. The prepared HC diet was subsequently supplemented with 60 g of galactose and 0.1 g of PCO or ATV, then mixed thoroughly with a spatula, followed by the addition of distilled water (~ 300 mL). The mixture was blended properly with a spatula and allowed to stand at RT for 4 hr. Finally, the mixture was re-agitated (~ 10 min) and subjected to freeze-drying to evaporate water. The freeze-dried sample was designated as HCHG enriched with PCO or ATV and named as HCHG+PCO and HCHG+ATV. Similarly, the HCHG diet was supplemented with 0.1 g each of PCO and ATV to prepare the HCHG-enriched PCO and ATV diet, abbreviated as HCHG+PCO+ATV. The prepared diets, HCHG and HCHG+PCO, HCHG+ATV and HCHG+PCO+ATV, were stored in a refrigerator (4°C) for further use.

Supplementary Figure S2

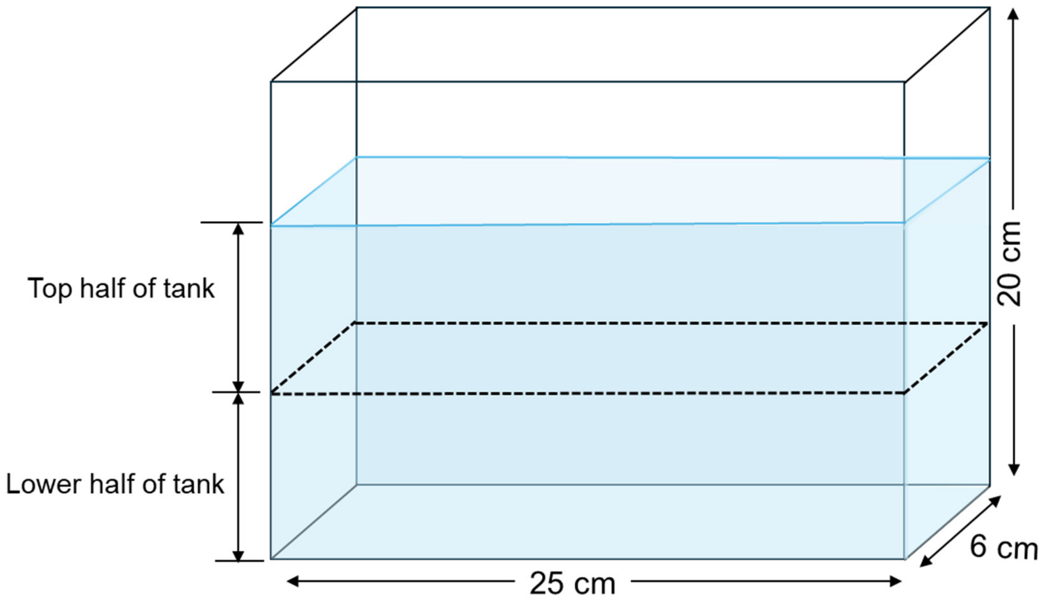

Supplementary Figure S2: Ray diagram of the open field water tank divided into two sectors (i.e., top half and lower half) using a dotted horizontal line.

Supplementary Figure S3

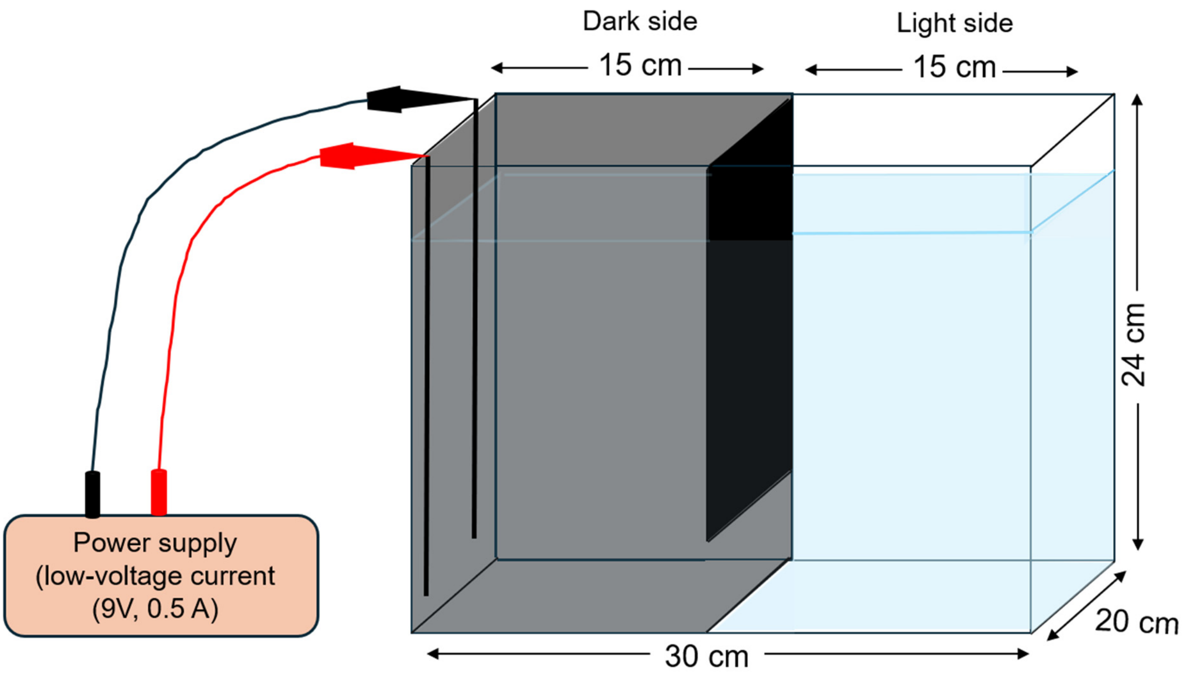

Supplementary Figure S3: Ray diagram of tank divided into two vertical halves (dark and light) used to perform passive avoidance test.
